# Pre-Hypertension and Its Determinants in Healthy Young Adults: Analysis of Data from the Korean National Health and Nutrition Examination Survey VII

**DOI:** 10.3390/ijerph18179144

**Published:** 2021-08-30

**Authors:** Insil Jang

**Affiliations:** Department of Nursing, Chung-Ang University, Seoul 06974, Korea; shili79@cau.ac.kr; Tel.: +82-2-820-5744

**Keywords:** hypertension, pre-hypertension, risk factors, adult nursing

## Abstract

The purpose of this cross-sectional study was to identify risk factors in the normotensive and pre-hypertensive group based on the blood pressure results of healthy young adults from the Korean National Health and Nutrition Examination Survey 2018. The participants were 2225 healthy young adults between the ages of 19 and under 45, excluding those with a diagnosis of hypertension or taking antihypertensive medications. Of the 2225 participants, the normotensive group was 1498 (67.3%) and the pre-hypertensive group 727 (32.7%). Determinants of pre-hypertension were analyzed using multiple logistic regression based on a complex sample design. Factors related to pre-hypertension in young adults were age, smoking, waist circumference, diabetes, anemia, cholesterol levels including HDL cholesterol, and uric acid levels. Pre-hypertension is a pre-stage that can prevent the morbidity of hypertension through lifestyle control, so its management is very important. Furthermore, a young adult is a stage in the growth and development of human beings, in which lifestyles such as healthy behaviors, eating habits, and exercise are fixed. Therefore, it is very important to improve lifestyles such as diet, exercise, and smoking cessation and to control risk factors in young adults who are at the pre-hypertension stage for health promotion. Continuous health examinations should be conducted for young adults, and education that can be practiced based on clinical data through this should be implemented for community health.

## 1. Introduction

As a global health problem, hypertension is closely related to cerebrovascular disease and cardiovascular disease, which are highly associated with mortality, so prevention and management are recognized as a very important problem for public health promotion. The prevalence of hypertension in Koreans has been steadily increasing over the past decades [1]. The prevalence of hypertension among adults over 20 in Korea is increasing to 25–29%, similar to that of the United States [2,3]. Early hypertension significantly increases the risk of stroke and cardiovascular disease before middle age. There is a much stronger association between stroke and blood pressure (BP) in Asians, including Koreans, compared to the US and European countries [3,4]. Asians are also more sensitive to salt and eat a high sodium diet (more than 4 g per day), which increases the risk of high blood pressure [4]. Recent studies have reported that strictly lower blood pressure lowers cardiovascular mortality [1]. Therefore, it is an important health and medical issue that the government must manage through policy for young adults through lifestyle control to prevent the progression to hypertension.

The 2018 Korean Society of Hypertension (KSH) blood pressure management guidelines in English were recently published in the August 2019 issue of Clinical Hypertension [5]. In the case of the American College of Cardiology/American Heart Association (ACC/AHA) Guideline, hypertension was changed to 130/80 mmHg or higher from 2017, but in Korea, it is still maintained at 140/90 mmHg or higher. A condition slightly higher than normal blood pressure and highly relevant to progression to hypertension is classified as pre-hypertensive in Korea. Pre-hypertension is a stage in which the relative risk of developing hypertension is doubled compared to normal blood pressure, and it is defined as a systolic blood pressure of 120–139 mmHg or a diastolic blood pressure of 80–89 mmHg [1,5]. Pre-hypertension is an early indicator of clinical hypertension and cardiovascular disease. The importance of this step is being emphasized because it can prevent the morbidity of hypertension through lifestyle changes. Therefore, it is essential to prevent the progression of hypertension through active nursing intervention and education that can detect risk factors related to pre-hypertension early and reduce them [6]. In addition, young adults have more autonomy over their decisions and begin to choose their own lifestyle choices, which can have a strong impact on their future health. In order to manage many chronic diseases that occur after middle age, such as diabetes and hypertension, including metabolic syndrome, a healthy lifestyle must be selected and applied from a young age.

Studies related to the risk factors of pre-hypertension were conducted both domestically and abroad, and based on the data of the National Health and Nutrition Examination Survey of each country, men, age, low income, smoking, drinking, high body mass index, and triglycerides, were presented [6,7,8]. However, most studies were conducted on all age groups or patients diagnosed with hypertension, and also, based on the Korean National Health and Nutrition Examination Survey (KNHANES), there was no case of dividing the pre-hypertensive group among healthy young adults [6,9,10,11,12]. There were few cases where clinical data were confirmed together with the results of physical examination or lab results such as blood and urine tests. The sample area of the KNHANES VII was extracted by the stratified multistage probability sampling design, which is a complex sample design method, in order to improve the representativeness of the sample and the accuracy of estimation. Therefore, when using the data from the KNHANES VII, a complex sample design that reflects weights according to the guidelines for use should be used to represent the entire Korean population [3]. In the literature using the existing Korean National Health and Nutrition Examination Survey data, biased results were presented using an analysis method assuming simple random sampling without using a complex sample design. Therefore, in this study, the KNHANES VII data of 2018 for the entire population were used as a complex sample design.

This study aimed to determine the prevalence of pre-hypertension and identify its risk factors in young adults using a representative sample of the Korean. The specific objectives of the present study were to (1) compare and analyze the difference between the sociodemographic, the anthropometric, biochemical, and clinical variables of the normotensive group and the pre-hypertensive group, and (2) identify risk factors influencing the pre-hypertensive group.

## 2. Materials and Methods

### 2.1. Study Design and Population

This study was a secondary analysis using the seventh KNHANES VII data (2016–2018) from the Korean Ministry of Health and Welfare. The KNHANES VII is a cross-sectional and nationally representative survey conducted by the Korea Center for Control and Prevention (KCDC) from 2016 to 2018. The survey used a stratified, multi-step probability sampling design in which each respondent was weighted to obtain equal probabilities so that the results were representative of the entire Korean population. According to the characteristics of cities and provinces and residential areas, the sample plots were first extracted by a two-step stratified random sampling method, and a certain number of households per survey district were sampled using the phylogenetic method within the extracted sample plots. The KNHANES consists of health interviews, health behavior and nutrition surveys, and health screening studies. The KNHANES VII is classified by year, and 2016 is divided into 1, 2017 is 2, and 2018 is 3. The KNHANES VII-3 was conducted for approximately 3518 household members aged 1 year or older by extracting a sample survey area in 2018. We requested the KCDC to use the survey results for research, submitted a data use plan, and posted the pledge on the KNHANES website. Informed consent was obtained from all participants. The study protocol was approved by the Korean Ministry of Health and Welfare.

This study analyzed 2225 adults who were younger than middle-aged (aged 19 to under 45), excluding those who were diagnosed with hypertension among adults who participated in the KNHANES VII-3, 2018. Participants were classified into either a normotensive group or pre-hypertensive group as per their blood pressure measurement results. Based on the criteria presented by the KNHANES, the pre-hypertensive stage was defined as systolic blood pressure (SBP) of 120 mmHg or more and less than 140 mmHg, diastolic blood pressure (DBP) of 80 mmHg or more and less than 90 mmHg. Normotension was defined as SBP less than 120 mmHg and DBP less than 80 mmHg.

### 2.2. Research Variables

#### 2.2.1. Sociodemographic Variables

The following sociodemographic characteristics were recorded: gender, age, occupation, marital status, education, household income, current smoking, weight change in 1 year, aerobic physical activity, monthly alcohol consumption, average sleep time in weekdays, the EuroQol five-dimensional (EQ-5D), the Patient Health Questionnaire (PHQ-9), and stress level. The household income level was classified based on the equivalent income (average monthly household income/$\sqrt{\mathrm{number}\;\mathrm{of}\;\mathrm{family}\;\mathrm{members}}$). Values in the lower 25% of the data were assigned lower status, and values in the subsequent three levels (25% each) were assigned lower middle, middle high, and high. Marital status was classified as yes or no when married, unmarried, without a spouse, or separated from a spouse due to death or divorce. Current smoking was categorized as heavy smoker for daily smoker, occasional smoker, past smoker, and non-smoker. Alcohol intake was based on the participants’ drinking patterns during the past month. Aerobic physical activity was divided into whether a week of moderate-intensity physical activity was performed for 2 h 30 min or more, high-intensity physical activity for 1 h 15 min or more, or a mixture of moderate-intensity and high-intensity physical activity (1 min of high intensity = 2 min of moderate intensity). The EQ-5D is a widely used survey instrument for describing health-related quality of life status [13]. To measure the subjects’ health-related quality of life, EQ-5D was used for exercise ability, self-management, daily activities, pain, and depression. The EQ-5D index value calculated by applying a weight to the measured value was used, and the closer to 1 point, the better the quality of life. Moreover, the PHQ-9 was used to check depressive symptoms. The PHQ-9 has the potential to be a dual-purpose tool for diagnosing depressive disorder and establishing grade depressive symptom severity with the same nine items [14]. The PHQ-9 Korean version was used and consisted of a total of 9 questions. Scores range from 0 to 27, ranging from 0 (never), 1 (for several days), 2 (more than half a day), to 3 (almost every day), for how often in the past 2 weeks it has occurred. There is a sensitivity of 88.0% and a specificity of 88.0% for major depression at points or higher [14].

#### 2.2.2. Anthropometric Variables

Height, waist circumference (WC), and weight were measured in units of 0.1 cm, 0.1 cm, and 0.1 kg, respectively. WC was measured end-tidal at the narrowest point between the lower border of the rib cage and the iliac crest using a handheld stadiometer ruler (Seca 225, Seca, Germany). The weight was measured using a calibrated balanced beam scale (GL-6000-20; G-tech, Seoul, Korea). Based on the measurements, body mass index (BMI) was calculated, and anthropometric measurements were similarly performed by well-trained examiners during the study period. BMI was classified as underweight if less than 18.5 kg/m^2^, normal if more than 18.5 kg/m^2^ to less than 23 kg/m^2^, and overweight if more than 23 kg/m^2^ to less than 25 kg/m^2^. In the case of obesity, BMI of 25 kg/m^2^ or more to less than 30 kg/m^2^ was classified into 1st stage, 30 kg/m^2^ or more and less than 35 kg/m^2^ in 2nd stage, and 35 kg/m^2^ or more in 3rd stage.

#### 2.2.3. Biochemical Variables

After an overnight fast for 12 h, a venous blood sample was taken and immediately sent to a central accredited laboratory for immediate plasma isolation by centrifugation. Fasting plasma concentrations of glucose, lipids, BUN, creatinine, and uric acid were measured using enzyme and hexokinase UV assay in a central laboratory using a chemical analyzer (Hitachi 7600-210; Hitachi, Tokyo, Japan). Hemoglobin and hematocrit were detected using the detection method (XN-9000; Sysmex, Kobe, Japan), and HbA1c was confirmed by high performance liquid chromatography (Toshoh G8; Toshoh, Tokyo, Japan). Urinalysis was analyzed using ISE (Hitachi 7600; Hitachi, Tokyo, Japan).

#### 2.2.4. Clinical Variables

Participants were asked to refrain from smoking or consuming caffeine before the measurements. Blood pressure was measured after resting for at least 5 min after arrival. Three consecutive measurements of systolic and diastolic blood pressure were obtained by a well-trained nurse using an appropriately sized cuff, bell on a standard stethoscope, and a mercury sphygmomanometer (Baumanometer; Baum, Copiague, NY, USA). Primary blood pressure was measured on the right arm, and secondary and tertiary blood pressure were measured at 30 s intervals. The average of secondary and tertiary blood pressure was used for analysis according to the guidelines for using raw data.

### 2.3. Ethical Considerations

This study was conducted after receiving permission for the use of data from the website of the National Health and Nutrition Survey. The KNHANES VII-3 data were reviewed and approved by the Institutional Review Board (Approval No. 2018-01-03-P-A) of the KCDC. Informed consent was obtained from all of the participants when the KNHANES VII-3 was conducted.

### 2.4. Data Analyses

The data were analyzed with the SPSS version 25.0 software (IBM Corp., Armonk, NY, USA) using a complex sample analysis, and the combined sample weight was calculated by multiplying weight by the ration of the number of survey units by year according to the KNHANES manual. For the data on the sociodemographic characteristics and health-related factors, categorical variables were presented as unweighted frequencies and weighted percentage. For the anthropometric, clinical, and biochemical variables, means and standard errors (SE) were calculated. The differences in sociodemographic characteristics, health behavior types, anthropometric, clinical, and biochemical variables of the normotensive group and the pre-hypertensive group were analyzed using the complex sample *x*^2^-test and t-test. Odds ratio (OR) and 95% confidence interval (CI) were calculated using a complex sample multivariate logistic regression to evaluate the risk factors affecting the pre-hypertension in terms of sociodemographic, health-related, clinical, and biochemical factors. Inferential statistical analyses were considered significant if the *p*-value was < 0.05.

## 3. Results

### 3.1. Sociodemographic, Health-Related, and Disease-Related Characteristics of Participants

Of the 2225 participants, the normotensive group was 1498 (67.3%) and the pre-hypertensive group 727 (32.7%). The sociodemographic characteristics of the normotensive and pre-hypertensive group were gender (χ^2^ = 256.92, *p* < 0.001), age (t = 85.02, *p* < 0.001), education level (χ^2^ = 14.58, *p* = 0.009), and household income (χ^2^ = 11.95, *p* = 0.044) were statistically significant (Table 1).

The difference in health-related and disease-related characteristics of the normotensive group and the pre-hypertensive group was found in the following variables. The variables, such as current smoking (χ^2^ = 74.15, *p* < 0.001), weight change in one year (χ^2^ = 27.03, *p* < 0.001), alcohol consumption (χ^2^ = 9.91, *p =* 0.009), PHQ (t = 15.45, *p* = 0.012), BMI (χ^2^ = 273.88, *p* < 0.001), hypercholesterolemia (χ^2^ = 57.76, *p* < 0.001), hypertriglyceridemia (χ^2^ = 82.14, *p* < 0.001), diabetes mellitus (χ^2^ = 106.70, *p* < 0.001), and anemia (χ^2^ = 18.79, *p* < 0.001) were statistically significant (Table 2).

### 3.2. Differences of Health Status of Participants between Normotensive and Pre-Hypertensive Group

Table 3 shows the differences in anthropometric, biochemical, and clinical variables between the normotensive group and the pre-hypertensive group. BMI (t = 126.79, *p* < 0.001) and waist circumference (t = 164.99, *p* < 0.001) were higher in the pre-hypertensive group than in the normotensive group, and there were statistically significant differences. Through blood and urine tests, fasting blood glucose, HbA1c, total cholesterol, triglyceride, BUN, creatinine, hemoglobin, hematocrit, uric acid, urine sodium, and urine creatinine were all significantly higher in the pre-hypertensive group. The normotensive group showed significantly higher HDL cholesterol than the pre-hypertensive group.

### 3.3. Factors Influencing the Development of Pre-Hypertension

As a result of performing a complex sample logistic regression analysis of variables with differences between the two groups, the variables affecting the occurrence of hypertension were age (40~ < 45 years; OR = 1.80, *p* = 0.030), education (high school; OR = 7.32, *p* = 0.003, current smoking (OR = 1.98, *p* = 0.002), hypercholesterolemia (OR = 2.02, *p* = 0.033), diabetes (OR = 5.26, *p* < 0.001), anemia (OR = 7.09, *p* <0.001), waist circumference (OR = 1.18, *p* = 0.004), HDL cholesterol (OR = 0.97, *p* < 0.001), and uric acid (OR = 1.19, *p* = 0.032) (Table 4).

## 4. Discussion

This study aimed to identify the prevalence of pre-hypertension and its risk factors in Korean healthy young adults. The high blood pressure rate among young adults in Korea is gradually increasing due to Westernized diet and stress, and it needs to be managed as a global health problem. Using the KNHANES data, the pre-hypertension of young people has been increasing since 2000, and other Asian countries such as China and Vietnam show similar trends [8,9,11,15,16]. Pre-hypertension frequently progresses to hypertension and is a public health problem that requires more attention as it also increases the risk of cardiovascular disease [6,17]. However, young adults tend to be overconfident about their health status, and in particular, an increase in blood pressure is easy to overlook because it is not associated with specific symptoms. Therefore, it is important to focus on the young people who can modify their lifestyle, especially the pre-hypertension group, where risk factors are found.

In this study, gender was not statistically significant as a risk factor for pre-hypertension. High blood pressure and cardiovascular disease are higher in men of all ages, but the prevalence is also increasing in postmenopausal women [11,18,19,20]. Gender did not act as an important factor in the rise of blood pressure in young adults before middle age, as there was no gender difference in the degree of recent work, social life, drinking, and smoking [6,15]. In this study, it was confirmed that the increase in blood pressure in healthy young people was caused by differences in social life and individual lifestyle rather than gender differences. However, the risk of pre-hypertension is increasing with the increase of age, and as a phenomenon related to aging, the same change is occurring in young adults [15,21]. Arteriosclerosis and vascular resistance are changing with aging, so it can be seen that young adults are no exception. Moreover, it was confirmed that the incidence of pre-hypertension differed by 4 to 7 times depending on the level of education, and it is thought to be related not only to differences in education, but also to the characteristics and intensity of occupations according to the level of education. In addition, current smoking was found to be a risk factor for pre-hypertension in this study in line with the previous results [6,22]. However, the risk level was lower than that of the study that confirmed all adults or the elderly, confirming the importance of management in young adults. Correction of the lifestyle of young adults should be emphasized according to age, not gender, and the emphasis on smoking cessation should be continued to reduce the prevalence of hypertension.

As a risk factor related to an increase in blood pressure, many existing studies have suggested a relationship with BMI [6,15,23,24]. However, weight change and BMI were not expressed as risk factors in this study; however, waist circumference was found to be a significant risk factor during physical measurement. Compared to middle-aged adults, young adults do not easily change their body weight due to their basal metabolic rate and physical activity. It is suggested that weight control is not unconditional in the management of blood pressure in young adults, as the weight usually increases after the middle-aged. However, the BMIs of the two groups were different, and the pre-hypertensive group was higher. It is premature to exclude it as a risk factor because it is important for young adults to maintain an appropriate weight through exercise and eating habits. Weight control is still important, and an increase in waist circumference is a risk factor that requires strict management for young adults [15,25,26]. In addition, the importance of waist circumference measurement as a more sensitive indicator than body weight should be emphasized. More research is needed to determine whether the effect of weight control and physical activity on blood pressure differs by age.

In this study, hypercholesterolemia, diabetes, and anemia were found to be risk factors for the pre-hypertensive group. Lab results showed differences in total cholesterol, triglyceride, and HDL cholesterol, and there was no difference in LDL cholesterol. Although the criteria for using medication such as statin to control cholesterol are based on LDL cholesterol, this suggests that young adults should be cautious about total cholesterol, triglyceride, and HDL cholesterol [6,27,28]. Previous studies have suggested a relationship between triglyceride, LDL cholesterol, and HDL cholesterol as the risk factors of pre-hypertension, but the exact factors were different. In this study, HDL cholesterol in young adults was identified as a risk factor in pre-hypertension, therefore dietary education focused on improving HDL is required. As showed in various studies, improvement of hyperlipidemia through various methods such as correction of dietary habits and exercise should be considered as an important intervention for preventing hypertension. Diabetes mellitus is closely related to an increase in blood pressure, especially after the age of 50, but this study suggests that it should be controlled as a risk factor for young adults as well [8,15,29]. Regardless of age, participants diagnosed with or suspected of diabetes must be strictly managed through follow-up with a healthcare provider. Furthermore, there was a difference between the two groups as supported by a study that confirmed a positive correlation between hemoglobin and systolic blood pressure [12]. However, as a result of the opposite, anemia was identified as a risk factor affecting the pre-hypertensive stage, so additional confirmation of other health conditions is required. It is necessary to confirm the association with hemoglobin through repeated studies in the future. Finally, uric acid level was higher in the pre-hypertensive group, and it was found to be a risk factor. In young Chinese adults, uric acid was positively correlated with sodium secretion and was confirmed as a risk factor for pre-hypertension [30]. This is related to the intake of salt, and it is a part that raises again the importance of controlling the eating habits to prevent a rise in blood pressure [4,6]. In particular, young adults tend to eat out frequently due to their active social life, which is easily overlooked due to their belief in health.

This study has several limitations as follows. This study was based on a cross-sectional survey, and secondary data were analyzed. There were restrictions based on data inclusion regarding diverse characteristics affecting the prevalence of pre-hypertension and its components. As there are limitations in explaining causality, a longitudinal study of a cohort approach is needed in the future. Second, some variables were measured by self-report format, careful interpretation is required, and repeated measurements are required. Nevertheless, this study used a multi-sample analysis method to identify risk factors related to pre-hypertension in young healthy adults. The strength of this study is that sociodemographic, anthropometric, biochemical, and clinical variables were identified together with the factors affecting the pre-hypertensive stage.

## 5. Conclusions

This study was attempted to provide basic data for developing a nursing intervention for the prevention of pre-hypertension by identifying risk factors that affect pre-hypertension and managing them. Factors related to pre-hypertension in adults between the ages of 19 and 45 confirmed through the KNHANES VII-3, 2018 were age, smoking, waist circumference, diabetes, anemia, cholesterol levels including HDL cholesterol, and uric acid levels. A young adult is a stage in the growth and development of human beings, in which lifestyles such as healthy behaviors, eating habits, and exercise are fixed. Therefore, lifestyle modification, such as diet, exercise, and smoking cessation, and risk factor control for young adults with pre-hypertensive stage, which can be altered, are very essential for health promotion. Through a national policy, continuous health examinations should be conducted for young adults, and education that can be practiced based on clinical data through this should be implemented.

## Figures and Tables

**Table 1:** Socio-demographic characteristics of the participants between normotensive and pre-hypertensive group.

Characteristics	Categories	Normotensive (*n* = 1498)		Pre-Hypertensive (*n* = 727)		χ^2^ or t	*p*
		U/F	W/F	U/F	W/F		
		N or M ± SE	%	N or M ± SE	%		
Gender	Male	512	50.7	495	49.3	256.92	<0.001
	Female	986	82.9	232	17.1		
Age (yr)		31.48 ± 0.30		33.25 ± 0.39		85.02	<0.001
	≤29	558	71.4	199	28.6		
	30~39	603	64.8	305	35.2	25.11	<0.001
	40~<45	337	58.1	223	41.9		
Occupation	Yes	976	64.2	528	35.8	11.14	0.073
	No	473	71.1	173	28.9		
	Others	49	59.7	25	40.3		
Marital status	Single	677	66.9	298	33.1	0.678	0.504
	Married	821	65.2	429	34.8		
Education	≤Elementary school	6	86.9	1	13.1	14.58	0.009
	Middle school	29	73.6	11	26.4		
	High school	488	61.6	292	38.4		
	≥College	926	69.0	397	31.0		
Household income	Lower	378	67.3	179	32.7	11.95	0.044
	Lower middle	363	62.1	196	37.9		
	Middle high	363	63.9	195	36.1		
	High	394	71.4	157	28.6		

Abbreviations: U/F, unweighted frequency; W/F, weighted frequency; EQ-5D, the EuroQol five-dimensional; PHQ, the Patient Health Questionnaire.

**Table 2:** Health-related and disease-related characteristics of the participants between normotensive and pre-hypertensive group.

Characteristics	Categories	Normotensive (*n* = 1498)		Pre-Hypertensive (*n* = 727)		χ^2^ or t	*p*
		U/F	W/F	U/F	W/F		
		N or M ± SE	%	N or M ± SE	%		
Current smoking	Heavy smoker	229	55.7	185	44.3	74.15	<0.001
	Smoker	55	59.0	39	41.0		
	Ex-smoker	217	55.7	157	44.3		
	Non-smoker	997	73.4	346	26.6		
Weight change (in 1 year)	No	808	68.5	357	31.5	27.03	<0.001
	Increasing weight	211	73.7	82	26.3		
	Decreasing weight	472	59.3	286	40.7		
	Others	7	79.7	2	20.3		
Aerobic physical activity	Yes	747	65.5	374	34.5	0.782	0.401
	No	702	67.3	327	32.7		
Alcohol consumption (frequency/month)	Yes (≥1/mo)	933	63.0	540	37.0	9.91	0.009
	No (<1/mo)	368	70.7	127	29.3		
Average sleep time, weekdays (min)		427.30 ± 2.69		423.64 ± 3.09		136.93	0.375
EQ-5D		0.97 ± 0.00		0.98 ± 0.00		392.06	0.484
PHQ		2.79 ± 0.12		2.28 ± 0.15		15.45	0.012
BMI	Underweight	119	89.8	16	10.2	273.88	<0.001
	Normal	785	79.1	207	20.9		
	Overweight	251	62.4	140	37.6		
	Obesity class I	267	51.1	254	48.9		
	Obesity class II	44	30.4	86	69.6		
	Obesity class III	7	17.0	19	83.0		
Stress level	None	154	64.8	76	35.2	5.36	0.421
	Low	837	64.9	413	35.1		
	Moderate	431	69.4	190	30.6		
	High	69	62.5	46	37.5		
Hypercholesterolemia	Yes	98	43.0	111	57.0	57.76	<0.001
	No	1334	68.9	581	31.1		
Hypertriglyceridemia	Yes	83	40.0	125	60.0	82.14	<0.001
	No	1093	70.7	426	29.3		
Diabetes mellitus	Yes	12	47.1	11	52.9	106.70	<0.001
	No	1486	66.3	716	33.7		
Anemia	Yes	128	83.5	26	16.5	18.79	<0.001
	No	1340	65.0	689	35.0		

Abbreviations: U/F, unweighted frequency; W/F, weighted frequency; EQ-5D, the EuroQol five-dimensional; PHQ, the Patient Health Questionnaire, BMI, body mass index.

**Table 3:** Differences of health status of participants between normotensive and pre-hypertensive group.

Factors	Categories	Normotensive (*n* = 1498)	Pre-Hypertensive (*n* = 727)	t	*p*
		M ± SE			
Physical examination	Height (cm)	166.96 ± 0.28	171.58 ± 0.34	499.99	<0.001
	Weight (kg)	63.39 ± 0.35	76.47 ± 0.73	104.87	<0.001
	WC (cm)	76.65 ± 0.29	87.04 ± 0.53	164.99	<0.001
	BMI (kg/m^2^)	22.63 ± 0.10	25.81 ± 0.20	126.79	<0.001
	Pulse (bpm)	54.95 ± 1.06	58.64 ± 2.49	23.54	0.183
	SBP (mmHg)	105.05 ± 0.25	124.89 ± 45	276.83	<0.001
	DBP (mmHg)	69.63 ± 0.19	85.74 ± 0.33	259.84	<0.001
Lab result	FBS (mg/dL)	91.22 ± 0.43	100.30 ± 1.23	81.207	<0.001
	HbA1c (%)	5.31 ± 0.01	5.55 ± 0.04	139.85	<0.001
	Total cholesterol (mg/dL)	184.02 ± 0.97	199.16 ± 1.77	112.44	<0.001
	Triglyceride (mg/dL)	103.67 ± 2.20	164.06 ± 6.77	24.24	<0.001
	HDL cholesterol (mg/dL)	54.13 ± 0.43	49.69 ± 0.50	9.44	<0.001
	LDL cholesterol (mg/dL)	122.37 ± 2.47	125.67 ± 2.59	48.46	0.380
	BUN (mg/dL)	12.86 ± 0.10	13.76 ± 0.14	96.95	<0.001
	Cr (mg/dL)	0.77 ± 0.01	0.87 ± 0.01	125.02	<0.001
	Hemoglobin (g/dL)	14.06 ± 0.05	15.31 ± 0.05	276.64	<0.001
	Hematocrit (%)	42.29 ± 0.14	45.59 ± 0.16	289.76	<0.001
	Uric acid (mg/dL)	5.12 ± 0.04	6.11 ± 0.06	98.83	<0.001
	Urine sodium (mmol/L)	107.53 ± 1.51	112.84 ± 2.10	53.70	<0.001
	Urine Cr (mg/dL)	185.17 ± 3.10	198.49 ± 4.16	47.70	0.003

Abbreviations: WC, waist circumstance; BMI, body mass index; SBP, systolic blood pressure; DBP, diastolic blood pressure; FBS, fasting blood sugar; HDL, high density lipoprotein; LDL, low density lipoprotein; BUN, blood urea nitrogen; Cr, creatinine.

**Table 4:** Multiple logistic regression analysis for risk factors of pre-hypertension prevalence.

Characteristics	Categories	Adjusted OR	
		OR (95% CI)	*p*
Gender	Male	1.02 (0.50–2.10)	0.960
	Female	1.0	
Age (yr)	≤29	1.0	
	30~39	1.14 (0.73–1.79)	0.046
	40~<45	1.80 (1.06–3.05)	0.030
Education	≤Middle school	1.0	
	High school	7.32 (1.90–28.15)	0.003
	≥College	4.80 (1.28–18.02)	0.002
Household income	Lower	1.0	
	Lower middle	1.11 (0.56–2.22)	0.416
	Middle high	1.84 (0.94–3.61)	0.442
	High	1.34 (0.66–2.72)	0.154
Current smoking	Yes	1.98 (1.24–3.14)	0.002
	No	1.0	
Alcohol consumption (frequency/month)	Yes (≥1/mo)	1.43 (0.80–2.60)	0.235
	No (<1/mo)	1.0	
Weight change (in 1 year)	No	1.0	
	Increasing weight	0.89 (0.51–1.55)	0.121
	Decreasing weight	1.36 (0.90–2.07)	0.141
PHQ		1.00 (0.94–1.06)	0.933
Hypercholesterolemia	Yes	2.02 (1.05–3.87)	0.033
	No	1.0	
Hypertriglyceridemia	Yes	1.66 (1.15–2.40)	0.059
	No	1.0	
Diabetes mellitus	Yes	5.26 (1.10–7.00)	<0.001
	Impaired fasting glucose	1.59 (0.92–2.75)	0.024
	No	1.0	
Anemia	Yes	7.09 (3.14–10.01)	<0.001
	No	1.0	
Physical examination	WC (cm)	1.18 (1.02–1.21)	0.004
	BMI (kg/m^2^)	1.03 (0.88–1.21)	0.716
Lab results	FBS (mg/dL)	1.01 (0.98–1.03)	0.658
	HbA1c (%)	0.83 (0.48–1.43)	0.493
	Total cholesterol (mg/dL)	1.00 (1.00–1.01)	0.222
	Triglyceride (mg/dL)	1.00 (0.99–1.01)	0.152
	HDL cholesterol (mg/dL)	0.97 (0.95–0.99)	0.001
	BUN (mg/dL)	0.99 (0.93–1.06)	0.825
	Cr (mg/dL)	0.80 (0.12–5.30)	0.818
	Hemoglobin (g/dL)	1.36 (0.84–2.22)	0.212
	Hematocrit (%)	1.02 (0.85–1.23)	0.816
	Uric acid (mg/dL)	1.19 (1.01–1.40)	0.032
	Urine sodium (mmol/L)	1.00 (0.99–1.00)	0.248
	Urine Cr (mg/dL)	1.01 (1.00–1.03)	0.389

Abbreviations: OR, odds ratio; PHQ, the Patient Health Questionnaire; WC, waist circumstance; BMI, body mass index; FBS, fasting blood sugar; HDL, high density lipoprotein; BUN, blood urea nitrogen; Cr, creatinine.

## Data Availability

Not applicable.
